# Evaluating the impact of a patient-representative model of support for women affected by cervical cancer

**DOI:** 10.1177/17455057251351415

**Published:** 2025-07-18

**Authors:** Elaine L. Kinsella, Elaine Kavanagh

**Affiliations:** 1 Department of Psychology, Centre for Social Issues Research, Health Research Institute, University of Limerick, Ireland; 2 Department of Psychology, University of Limerick, Ireland

**Keywords:** patient and public involvement, patient representation, patient advocacy, healthcare reform, patient representative model

## Abstract

**Background:**

In the aftermath of high-profile healthcare system failures, patient representative groups can emerge as key contributors to support, reform, and accountability. Following the identification of failures in Ireland’s CervicalCheck screening programme in 2018, the 221+ Patient Representative Group (commonly known as 221+) was established to support affected women and families.

**Objectives:**

The present research aimed to assess the impact of the 221+ group and associated patient representatives in supporting women and influencing the delivery of healthcare.

**Design:**

An independent research team conducted a two-phase qualitative study, gathering perspectives from a range of stakeholders involved in statutory, non-statutory, and voluntary healthcare sectors in Ireland.

**Method:**

Interview data collected from key stakeholders (phase 1, *N* = 15) and qualitative survey responses from medical and healthcare professionals (phase 2, *N* = 86) were analysed separately using reflexive thematic analysis.

**Results:**

Findings support the value of a patient-representative model in providing support and advocacy for women affected by cervical cancer and their families, while also highlighting important considerations such as sustainability, integration with healthcare systems, and the need for inclusive representation.

**Conclusion:**

This study provides a case example of patient advocacy in action, offering transferable insights and strategies to inform patient-centred care and healthcare reform in other settings.

## Plain language summary

In 2018, systemic failures in Ireland’s CervicalCheck screening programme were brought to light following Vicky Phelan’s court case in April 2018. As a result, the 221+ patient support group was established to provide information, advice and support to the 221+ women and families directly affected. The present research was undertaken to evaluate the extent to which the 221+ patient support group has achieved its aims and, in doing so, influenced healthcare delivery in Ireland. To this end, fifteen stakeholders, from various statutory and voluntary sectors were interviewed, and 86 medical and health professionals on the gynaecological health patient pathway completed an online survey. Key findings indicated that 221+ provided a supportive service and was a positive advocate for women and women’s healthcare, while also highlighting key areas for learning and future development. The research provides insights into how 221+ and other patient representative groups might move forward in supporting women’s health and health services.

## Introduction

Medical errors represent a significant challenge in healthcare systems worldwide. For patients and families, errors have the potential to not only cause immediate physical, emotional and psychological harm, but also can have long-term social and financial consequences.^1^ While the focus often falls on mistakes made by individual healthcare providers, systemic failures have also played a major role in some of the most devastating healthcare scandals globally. Examples include the Mid Staffordshire NHS Trust Scandal (U.K.),^2^ the Chhattisgarh sterilisation deaths (India),^3^ and various contaminated blood scandals (e.g. U.K., France, Japan, and others).^4,5^ Importantly, the way these events are handled can exacerbate the harm experienced by those affected.^1,6^

Systemic medical errors not only cause immediate harm but often lead to wider societal repercussions, including the creation of groups that seek to support those affected, and/or seek accountability and reform. In Ireland, for instance, the 221+ patient representative group was established in 2019 following failures in the CervicalCheck screening programme. This group was named after the 221 women who were initially identified as being affected by systemic failures in the screening programme for cervical cancer. Following an audit of CervicalCheck test results, many of these women were not informed of incorrect smear test results, some of whom had developed cervical cancer. The original purpose of the 221+ patent representative group was to support women and families directly affected by failures in the screening programme. In 2024, the group (commonly referred to as 221+) continues to exist and works to ensure that the voices of those affected are heard in discussions about healthcare policy, oversight and reform, in addition to supporting members.

The CervicalCheck failures highlight the complexity of medical errors, which often extend beyond individual negligence to involve systemic breakdowns in communication, transparency, quality assurance and oversight. An independent scoping inquiry, led by Dr Gabriel Scally, into the CervicalCheck Screening national programme, a series of recommendations have been implemented to improve screening services in Ireland.^7,8^ When commenting on the reformed CervicalCheck screening programme, Dr Scally^7^ stated that ‘women can have confidence in and should take full advantage of the cervical screening programme. It has saved many women’s lives and will continue to do so’. Central to these reforms was feedback from members of 221+, whose experiences shaped many of the changes. However, research has yet to examine the effectiveness of the 221+ group in representing patients and supporting women and their families, which is the focus of this study.

### A changing landscape for healthcare: patient and public involvement

**Figure 1.**
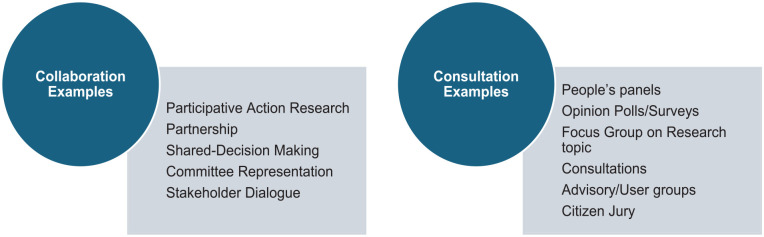
Consultation versus collaboration.

Patient and public involvement (PPI) is considered a central aspect of health reform agendas in European health systems.^9–11^ The development of strong PPI reflects a recognition of the value that patients and the wider public bring to healthcare, including service planning and delivery.^9^ PPI can vary from participation in public polls, surveys, seminars, workshops, or focus groups to membership of steering groups, service development or monitoring groups for clinical practice guidelines.^12^ However, research has suggested that professionals can sometimes resist and even feel threatened by the notion of active patient participation.^10–13^ Indeed, while some would argue that patient and public representatives promote a civil society, it is frequently countered that such representatives speak to the experiences of a minority or niche group and are therefore, not representative or provide objective views that would be of benefit to the greater population.^10,13^ Essentially, the promotion of PPI is patchy and slow and frequently represents the lowest level of involvement, that is, consultation rather than collaboration (see Figure 1 for examples of consultation versus collaboration).^11^

Nonetheless, patient safety campaigners are now part of a recognised social movement^14^ and one that has learned from other civil movements highlighting inequality and power. Rights-based groups first emerged in healthcare as far back as the 1970s^11^ and emphasised the limitations of the biomedical model in promoting health and illness with calls for a more shared model of treatment and decision-making.^11^ Past paradigms of ‘normality’ were challenged by such social movements, for example, the gay rights movement, which had a profound impact on involvement in health. There was a protracted international campaign to have homosexuality removed from the World Health Organisation’s *International Classification of Diseases* and the *U.S. Diagnostic and Statistical Manual of Mental Disorders*.^15^ This campaign represented an important landmark as it highlighted how definitions of ‘illness’ can be challenged by political action and accepted scientific ‘truths’ can be undermined and altered on foot of public protest.^16^ As a result, rather than a clearly defined phenomenon, PPI can be viewed as a collection of practices which often take place in a context where the distribution of power is being contested.^16,17^

More recent manifestations of challenges to healthcare provision have come from campaigns by patients who had been harmed during their care, including actions taken by patients’ relatives.^11^ Arguably, these more contemporary campaigns were reinforced by those broader arguments which emerged in the 1970s. Ongoing issues concerning health service delivery remain relevant in modern society and illustrate how the balance of power seemingly favours the organisation or system rather than collaborative approaches with patients and the public.^11,18,19^

In a context where patient and public representatives are increasingly viewed as central to the delivery of good patient and family-centred care, finding effective ways to evaluate PPI is important.^11^ Several factors make such evaluations a challenge including the need for a shared understanding about how PPI is conceptualised and measured as well as difficulties in isolating involvement from other factors that may have influenced change.^20^ Frameworks used for evaluation tend to be situated within a positivist empiricist approach, using quantitative surveys to measure patient experiences,^21^ the outcome of which does not necessarily lead to quality improvements.^22^ Qualitative approaches have been proposed as a method with the potential to capture real-world stories, with the additional potential capacity to generate interest among health service providers and noted as an under-exploited method of evaluation.^21^ Therefore, efforts to evaluate PPI approaches need to consider a range of ways of generating data, including qualitative approaches, to include a wider range of expertise and experiences.

In the present research, we focus on the 221+ patient representative group, which consists of and represents a subset of women in Ireland with cervical cancer, affected by past systemic failures in the CervicalCheck screening programme. In essence, by joining 221+, those women became part of a PPI process.

## The present research

The available literature suggests that most medical and health professionals and patient representatives agree that a supportive patient organisation is advantageous to the representativeness of the ‘patient voice’.^10^ To date, no research has been undertaken into the influence of a patient-representative group, such as 221+, in representing women and families to support each other and to advocate for healthcare reform.

To capture a breadth and depth of perspectives, the present research developed a two-phase, multi-method qualitative research design to assess the work and impact of the 221+ patient representative group to ascertain the extent to which the group, including its patient representatives, influenced change in the provision of healthcare to women affected by cervical cancer in Ireland, including information as to the effectiveness of support provided to women and their families in the aftermath of a healthcare system failure.

This research draws on qualitative data from a survey, which was distributed to medical and health professionals and qualitative interview data with key stakeholders collected as part of an evaluation of the 221+ patient representative group. This study aimed to assess the impact of the work completed by the 221+ patient representative group in meeting the needs of its members, specifically, women and families impacted by failures in the CervicalCheck system in Ireland.

## Methodology

A participatory methodology^23^ was employed by the research team in recognition that the 221+ patient representative group is a small and unique in its representation of members impacted by systems failures in CervicalCheck in Ireland, with limited funding and the present study could play an important role in strengthening the group’s familiarity with research and evaluation.^24^ The participatory approach included co-design of the evaluation methods, survey distribution and identification of key participants for interview through those at the senior management level in the 221+ group.

## The research approach

The research was completed in two separate phases, from October to December 2023, with two distinct samples to capture the breadth and depth of perspectives relevant to the research aims and objectives. The first (phase 1) consisted of semi-structured interviews with key stakeholders (*N* = 15) from a range of groups and organisations that have worked with or interacted with 221+ patient representative groups since its inception in 2018. Phase 2 (*N* = 86) of the research aimed to acquire the anonymous views of a purposive sample of health and medical professionals in Ireland about the work of the 221+ patient representative group. Reflexive thematic analysis was deemed the most appropriate type of analysis for both phases of this multi-qualitative study in the context of the underlying paradigmatic assumptions, namely, a constructionist epistemology using an experiential orientation to data interpretation.^25^ In addition, the use of reflexive thematic analysis allowed the data of both phases to be analysed in a manner which respected the subjectivity of participants’ experiences while acknowledging the reflexive influence of researcher interpretations.^25^ The Reflexive Thematic Reporting Guidelines (RTARG) represent a framework to assess the quality of reflexive thematic analysis.^26^

### Phase 1: interviews with key stakeholders

Participants for phase 1 were identified following detailed conversations and planning between the 221+ group and the research team, culminating in a stakeholder map which sought to capture a diverse range of perspectives. Interview participants were required to have in-depth knowledge about 221+ to meet inclusion criteria. Invitations to participate in phase 1 were facilitated by 221+, and the identities of those that responded to the call to participate remain known only to the research team. Participants were associated with both voluntary and statutory sector healthcare organisations in Ireland, such as health and medical professionals, as well as those involved in advocacy, development of policy, health promotion, charity/NGO employees, and cancer survivors.

Participants were interviewed to gain insights and perspectives about 221+. A semi-structured interview schedule was tailored to capture areas in which 221+ had been most effective and included questions about how 221+ might develop into the future (interview schedule available in Supplemental Material). The use of a semi-structured interview schedule ensured participants were asked the same core questions which enhanced uniformity in questions asked of participants representing a consistent method of data collection. In addition, this approach allowed a degree of flexibility for clarifying questions. At the final stage of the interview process, all participants were invited to share any additional comments or insights they wished to add, allowing for a more comprehensive capture of key information. Written consent was provided prior to interviews, and additional oral consent was obtained at the start of all interviews.

#### Data analysis of interviews

**Figure 2.**
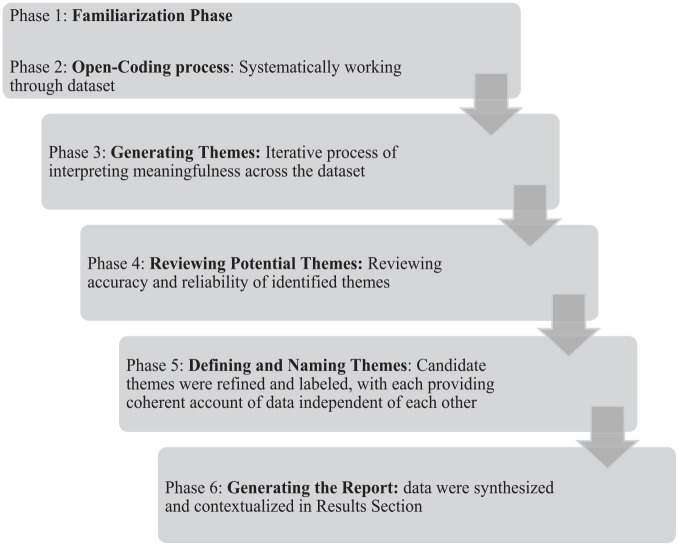
Stages of analysis.

Interviews were digitally recorded and transcribed by the research team. An important aspect of data analysis was to reflect stakeholder accounts as faithfully as possible while also accounting for the reflexive influence of the researcher’s interpretations.^25^ Reflexive thematic analysis was considered the most appropriate type of analysis in the context of the underlying paradigmatic assumptions of the present research, namely, a constructionist epistemology using an experiential orientation to data interpretation.^25^ Its application facilitated a reflection on interview participants views about 221+ while also acknowledging the influence of the researcher’s interpretation of their accounts.^25^ The analysis was informed by the evaluation questions and transcripts were coded within NVivo software programme. Data were analysed inductively, and a six-stage process was employed (see Figure 2), which involved a process of immersing in the data and also distancing from it, to facilitate both reflection and analysis.^27^ One author (Ekav) completed steps 1–3 before reviewing in detail with author Ekin, and together the research team completed steps 3–6, analysis remained an inductive and iterative process of discussion and reflection.

Specifically, interviews were manually transcribed, and each transcript was read several times. The data were input into NVivo software and codes were generated through systematically working through the entire data corpus (phase 2). Phase 3 began when all relevant data items were coded and the focus shifted from the interpretation of individual items within the dataset to the interpretation of meaningfulness across the dataset, a process during which there was active immersion in the data and also periods of distancing from it to allow time for reflection to develop.^27^ Throughout phase 3, we focused on theme interpretation and how codes communicated meaningfulness in answering the research question.^25^ In phase 4, both authors reviewed the accuracy and reliability of identified themes and analysis remained iterative on two levels. First the candidate themes and codes that informed them were reviewed to ensure they contributed to the overall narrative and second, candidate themes were reviewed in relation to each other to ensure they answered the research question.^25^ Themes were then refined and labelled, and phase 6 involved the generation of the report which subscribed to the recommendations of Braun and Clarke where the data was contextualised and synthesised with the available literature as reported in the section ‘Results’.

Through this research approach to analysis of interview data, we determined that data saturation was reached through a careful and iterative process of analysis, where no new significant themes or patterns emerged. In line with Malterud et al.’s^28^ concept of ‘information power’, we considered both the size of the sample and the richness of the interview data in evaluating saturation. Information power suggests that saturation is reached not only through the number of participants but also through the depth and relevance of the data generated by each participant. Our sample, which consisted of 15 interview participants, provided a rich variety of perspectives that were central to the research question. The authors collaboratively analysed the depth of meaning in the interview data, and the saturation point was reached when no new themes or ideas that significantly impacted our understanding of the research question were interpreted.

### Phase 2: survey of health and medical professionals

To meet the inclusion criteria for the second phase of the study, respondents were required to be over 18 years of age and be employed in the medical and health professional sector. No incentives were offered for participation. The survey was distributed via Qualtrics survey software and was available online from 20 November 2023 until 23 December 2023. Consent to participate was required from all survey respondents prior to survey completion. It was circulated to a purposive sample of health and medical professionals who women encounter as part of the cervical smear process. It was hoped that the anonymous information obtained from professionals along this specific patient pathway would serve to further an understanding of clinicians’ views and potentially generate opportunities to build stronger communication bridges between patients and health services.

Open-ended qualitative survey questions focused on health and medical professionals’ views about the work of 221+. The survey included three basic demographic questions relating to gender, occupation and age range and five qualitative questions which included questions about their knowledge of 221+ and views about how 221+ might contribute to women’s healthcare in the future (survey questions are available in Supplemental Material). Participants were required to indicate their consent before progressing to survey questions.

### Survey sample

The survey received 86 completed responses, which were included for analysis. Key demographic characteristics are summarised in Table 1.

**Table 1.** Summary of survey participant demographics.

Participant characteristics	Summary
Gender	Female: 75 Male: 7 Prefer not to say: 4
Age (ranges)	35–45 years: 21 45+ years: 60 Prefer not to say: 5
Employment	Healthcare professionals: 82 Care worker: 2 Prefer not to say: 2

In addition, in responses to a question about whether the 221+ patient representative group had a positive impact on healthcare in Ireland, 44 respondents indicated their agreement (51%), with 11 (13%) respondents indicating disagreement and a further 31 respondents choosing to omit a response to the question.

#### Data analysis of survey responses

Survey responses were collected via Qualtrics survey software and then imported into NVivo software package for analysis. Demographic data were analysed in NVivo, and frequencies and attributes were generated within the software package. In the context of the small sample size, the use of NVivo software was used to facilitate the synthesis of qualitative responses to survey questions with demographic information, which generated a broad understanding of the sample group.^29^ Qualitative survey responses were analysed inductively and separately from interview data and informed by the evaluation questions and a six-step approach to analysis was taken.^27^

## Results

This section explores the effectiveness of the 221+ Patient Representative Group in representing a subset of women affected by past systemic failures in the CervicalCheck programme and examines whether their work has influenced healthcare provision in Ireland based on data collected during phase 1 and 2 of this research. The findings are framed around evaluation questions which examine perceptions of 221+ past actions, current endeavours and views about future development opportunities.

Findings are summarised, with evidence provided through the inclusion of confidential extracts from both survey responses and participant interviews. While survey respondents were anonymous and not assigned an identifier, to maintain the anonymity of interview participants, the names of participants are not used but are instead assigned a number and are reported as Participant 1 (P1), Participant 2 (P2). In some cases, information within a particular quote has been omitted to ensure the individual remains anonymous, and in those cases, the omitted lines have been indicated using three dot ellipses [. . .] (Table 2).

**Table 2.** Themes and associated subthemes.

Themes	Subthemes
‘A lifeline I didn’t know I needed’: The gift of support through life-changing crisis.	Presence of ‘patient voice’ improved healthcare services for women Importance of peer support through crisis Recognising cervical cancer stigma
‘An enormous cost and enormous benefit’: Advocacy needs to have a very broad reach	Persistent and consistent communication and advocacy
A crossroads of opportunities: ‘They could move into a space where they support all women with cervical cancer’	Speak for all women with cervical cancer Build trusted bridges between medical and health professionals and those using healthcare services Extend sphere of influence to bring ‘lived experience’ into educational arenas

### Theme 1: ‘a lifeline I didn’t know I needed’: the gift of support through life-changing crisis

This section considers the impact of 221+ on healthcare delivery since its formation in 2018, including supporting women and families as they navigated life following a systemic failure in the CervicalCheck screening programme.

#### Presence of ‘patient voice’ improved healthcare services for women

Both interview and survey participants credited 221+ with improving healthcare for a subset of women in Ireland impacted by past systemic failures in the CervicalCheck programme. Survey respondents, for example, pointed to 221+ supporting and empowering the women and families affected: ‘(221+) remained committed to having women and families’ voices and stories heard at national decision-making level’, and advocating for vaccinations against the human papillomaviruses (HPV) virus: ‘They (221+) have advocated for vaccination against HPV which will save a lot of lives’. Interview participants concurred and provided greater detail about the significance of the ‘patient voice’ in the development of healthcare delivery by ensuring patient perspectives were consistently considered in decision-making forums and service-user feedback, leading to more effective and beneficial outcomes: ‘We’ve seen a significant investment by government in women’s healthcare really since the establishment of 221+. . . I think that they’ve really put the voice of women more centrally into government decision making’ (P7).

Also, 221+ was credited with advocacy efforts that led greater levels of investment by government in women’s healthcare, and a greater openness to talking about the symptoms and treatment of cervical cancer. 221+ was described as yielding considerable influence in relation to the Patient Safety Act (2023), supporting open and honest disclosure amongst patients and health services providers or practitioners in Ireland. The input of 221+ was seen to have provided critical input on how screening programmes are conducted, focusing on efficacy, transparency and sensitivity to the needs of patients.

Conversely, one interview participant alluded to how the actions of 221+ have led it to become accused of generating a lack of trust in CervicalCheck in Ireland, such as P10: ‘I think some people would blame 221+ and the families involved for, I suppose, engendering a lack of trust in the screening services’. In the survey sample, 13% (*n* = 11) of medical and health professionals reported the view that 221+ had not had a positive impact on healthcare in Ireland. While the survey did not seek further elaboration from medical and health professionals, it remains pertinent that it was expressed, albeit by a minority of respondents. Nonetheless, both survey and interview data pointed to a continued need for emphasis on the patient voice in healthcare service delivery:

The voice of 221+ is needed. It really adds a lot in the area of women’s healthcare around patient voice and patient involvement, and it would be my hope that it continues to work in those areas to make sure that women are empowered when engaging with the health system and that that will ensure that all of us are the owners of our health and that it’s not someone else who makes decisions for us. (P7)

These sentiments correspond with existing literature about the positive impacts of patient representatives in healthcare service delivery and reform.^10,21,30^ The input of 221+ patient representatives was welcomed by the vast majority of research participants, with participant interviews expressing a caveat: 221+ need to ensure that patient representation remains a central feature and not become a tokenistic symbol of true PPI, a point which has also been raised as a concern in the existing literature.^11,31^

#### Importance of peer support through a life-changing crisis

In interview and survey responses, participants recognised that 221+ developed a crucial social support network for women and families impacted by systems failures in the CervicalCheck, including social and emotional support for those who chose to take a litigious route, which was accepted as representing an acute life challenge. Relief was expressed that such an organisation existed to support women and families impacted, with an assumption expressed that 221+ will continue to provide support to women and families into the future:

It is wonderful knowing that they are actively working behind the scenes to ensure the support for patients and their families, and all future women that will be using the CervicalCheck screening programme.

Interview participants described how 221+ had provided crucial social, emotional and psychological support which was effective and provided an opportunity for those impacted to meet similarly situated others, as P6 explains:

The 221+ group have been a lifeline. It has been a lifeline I didn’t know I needed. . .I didn’t know who to turn to, I didn’t know anything about anything. . . So then, to be able to get in touch with the 221+ group was such a lifeline because you were able to meet like-minded people. People who just get you. (P6)

This finding is consistent with existing research on how peer support buffers negative emotional experiences. The available literature has highlighted how the benefits of in-group support can diminish stress experiences and enhance well-being, allowing those impacted to interpret a significant life challenge as more manageable.^32–34^

#### Recognising cervical cancer stigma

An aspect noted in interviews but not referenced by medical and health professionals in surveys was cervical cancer stigma. Some interview participants referred to the importance of the 221+ group’s commitment to advancing the development of healthcare delivery for women as particularly critical due to a stigma associated with cervical cancer. 221+ actions were described as stimulating a greater openness and factual knowledge about the disease. Some interview participants referenced a continued stigma associated with a cervical cancer diagnosis due to connotations of sexual promiscuity, which led women to withhold their diagnosis from their family, friends and their community as they feared judgement:

It wasn’t known that they’d cervical cancer. And largely that was because of the stigma of cervical cancer and their feeling that they would be judged morally because of their cancer. (P14)

Interview participants cited views about how 221+ was in an optimal position to represent those that chose to avoid disclosure of their illness due to associated stigma. Stigma associated with cervical cancer was cited as a systemic barrier to equitable healthcare that requires continued research, education and advocacy to address.

### Theme 2: ‘an enormous cost and enormous benefit’: advocacy needs to have a very broad reach

Both survey respondents and interview participants noted how 221+ approached patient representation collaboratively and persistently. The group were not seen to wait to be consulted about changes implemented in cervical screening and healthcare delivery for women in Ireland but rather were proactive in seeking change.

#### Persistent and consistent communication and advocacy

Survey and interview participants identified effective strategies used by the 221+ group in seeking to improve healthcare for women: persistence in advocacy which manifested through consistent and effective communication across statutory, voluntary, and political arenas.

Survey respondents recognised 221+ as an organisation whose persistence resulted in increased awareness about the need for transparency and openness in healthcare service delivery. Specifically, in cancer screening for women in Ireland, 221+ were acknowledged as a group that consistently sought ongoing assurances to prevent shortfalls in service delivery, requiring accountability at all decision-making levels, and crucially, ensuring the patient voice via patient representatives continued to influence and inform policy decisions. Medical and health respondents pointed to how 221+ had positively impacted awareness about the importance of cervical screening and encouraged women to become proactive in their own health, thus challenging potential paternalistic models of healthcare:

[The work of 221+] reinforced to me as a nurse that the test is only as good as the person carrying it out, and the person interpreting the results and the need to advise clients to always follow their gut instinct i.e. if they feel that things are not right even though they have been reassured by professionals to seek further advice.

Similarly, interview participants viewed 221+ and its patient representatives as having communicated consistently and effectively across important sectors to achieve its goals and objectives, including the use of a media focus, an aspect on which 221+ efficiently capitalised to ensure recommendations arising from scoping enquiry and reports (see Scally^8^) were implemented in full:

They [221+] realised early on that it wasn’t just about a report being published. It was about holding the state agencies to account to ensure that they were fully implemented. (P7)

Both survey and interview participants viewed 221+ as an agency with the ability to successfully, and consistently, communicate across all sectors resulting in their garnering all-party (i.e. political party) support across the political spectrum in Ireland which yielded considerable influence and ultimately ensured consistent progress towards improvements in the delivery of healthcare to women in Ireland:

It was one of those few processes that got all-party support across the Dail, across the Oireachtas. . .I think the influence that they had was that it didn’t get politicised. . .everybody wanted to see this fixed and the harm that was done acknowledged. (P15)

Interviews with stakeholders provided some deeper insights and referenced an awareness that sustained persistence and consistency in patient representation came with a cost (physical, social, emotional, financial, and temporal):

All of these things come with enormous cost to them. . . . Have come with an enormous cost to them, and enormous benefit to them as well, I’m sure, in terms of the respect that they’ve quite rightly gained and that they’re feeling they’re doing the right thing. But, you know, there’s a downside to it for them as well. (P14)

### Theme 3: a crossroads of opportunities: ‘they could move into a space where they support all women with cervical cancer’

While 221+ was born in a crisis, the contributions to healthcare were acknowledged by both interview participants and survey respondents with many suggesting the continued development of areas associated with healthcare education, building trust and for 221+ to expand its influence to continue to positively impact women’s healthcare in Ireland.

#### Representation for all women with cervical cancer

At present 221+ represents a subset of women in Ireland that were impacted by past systemic failures in the CervicalCheck screening programme. While both medical and health professionals and interview participants credited 221+ with improving healthcare provision for those affected, only interview participants spoke about their hope that 221+ might be supported to expand their remit to include *all* women impacted by a cervical cancer diagnosis albeit without losing focus on their overarching goals: ‘I guess that is my hope for 221+ is that they could move into a space where they support. . .all women with cervical cancer’ (P8).

#### Build trusted bridges between medical and health professionals and those using healthcare services

Survey respondents referenced a need for a more collaborative approach between healthcare professionals and the 221+ patient representative group to promote clear communication around the benefits of cancer screening, vaccination programmes and towards restoring trust in healthcare service delivery. Indeed, survey responses from medical and health professionals referenced how they experienced collateral harms on foot of misinformation and poor communication which resulted in a lack of trust in healthcare services, specifically concerning cervical cancer screening: ‘Very difficult in dealing with aggressions, nearly to a point of leaving the health care service’. Medical and health professionals did *not* attribute these experiences to any actions taken by the 221+ group but emphasised the need for a more collaborative approach to mitigate collateral harms.

Interview participants acknowledged the negative impact system failures in CervicalCheck had on medical and health professionals’ women encountered along the ‘patient pathway’. In this context, the need for a restoration of trust between medical and health professionals and those women and families impacted by cervical cancer was perceived by those interviewed as crucial. Interview participants articulated a view that the 221+ patient representative agency as well placed to play a pivotal role in dispelling misinformation, misrepresentation and enhancing the significance of the ‘patient voice’ between those professionals encountered along the ‘patient pathway’. The available literature highlights the importance of promoting patients to become empowered to facilitate the establishment of trust in arenas where considerable power differentials exist between medical providers and patients.^21^ The establishment of trust requires a genuine collaborative approach between patients and medical and health professionals.^11^ Restoration of trust was cited by participants as a critical factor and key pathway towards the development of stronger partnerships between healthcare providers and those who use services. ‘They (221+) also played a very key role. . .of building that process of trust or re-building the process of trust’ (P15).

However, building trust and more collaborative networks was described as a potentially challenging road, but an important endeavour. For example, some medical and healthcare professionals cautioned against the 221+ group becoming known as a group that supported more litigious actions, an aspect which may have resulted in a reduction of trust (however unintentional) between CervicalCheck and women availing of cancer screening. One medical or healthcare professional described how a loss of trust in healthcare delivery and ongoing actions of a litigious nature had negatively affected patients and their families in both psychological and social ways:

I have cared for members of the group in their palliative stages. This has invariably been challenging due to the loss of trust in the healthcare system and the professions within and by where litigation is ongoing this comes with very significant impacts on the patient and family’s journey psychosocially, often with increased anguish and a heightened anxiety around the legacy left behind if these issues are left open and/or unresolved.

#### Extend sphere of influence to bring ‘lived experience’ into educational arenas

Medical and health professionals suggested that the 221+ group might consider a targeted educational campaign around screening services through engagement with schools and third-level institutions, to influence and emphasise messages about the importance of cancer screening programmes and vaccination programmes (‘[221+ should] engage in working with young people, schools, colleges, educating and advising on the benefits of proactively engaging with cancer screening’). This was particularly pertinent in the context that a proportion of medical and health professional respondents were unaware of the 221+ agency before receiving a request to participate in research. This led to health professionals stressing a need for the 221+ group to become more visible through greater engagement with educational programmes and suggested growing its online presence and sourcing effective public engagement strategies.

Interview participants suggested that 221+ consider bringing a ‘lived experience’ into educational settings,^35^ specifically targeting medical and health professionals, in awareness campaigns to highlight the importance of screening and vaccinations and finally as a possible pathway towards diminishing perceptions of the stigma associated with the disease^36^: ‘They have good vignettes, good real-life stories, experiences of what people had, to make it real, you know, for people, it’s powerful’ (P15).

## General discussion

An analysis of survey and interview data underscore the significant impact 221+ has made on the healthcare landscape for women in Ireland. The findings highlight how the patient representative group persisted in a collaborative approach with all pertinent agencies across voluntary, statutory and political arenas to ensure that the ‘patient voice’ became a central focus across the ‘patient pathway’. The work of 221+, as recounted by research participants, has resulted in improved healthcare services, specifically, cervical screening services, for women in Ireland. These findings add to the existing literature about the effectiveness and concrete benefits of meaningful collaboration with the ‘patient voice’ rather than a more two-dimensional consultative approach.^11^

Interestingly, despite reports pointing to medical and health professional reluctance to treat women associated with the 221+ patient representative group,^7^ there were significant similarities between key interview participants and health professionals’ views about the 221+ group’s key contributions, evidenced strengths and future areas of development. The positive perceptions about the actions of the 221+ group emanating from medical and health professionals along the ‘patient pathway’ suggest an openness towards meaningful and collaborative PPI. These research findings point to how meaningful PPI can assist in the restoration of trust between healthcare providers and those who use healthcare services. Collaborative engagement may also serve to mitigate misinformation and poor communication which this research found caused medical and healthcare staff to experience collateral harm (‘dealing with aggressions’).

An additional factor which compounded the harm experienced by systems failures in the CervicalCheck screening programme was stigma, a unique feature of this cancer (and perhaps other forms of gynaecological cancer) and specifically affects women, transgender men and non-binary people assigned female at birth. Notably, this area was referenced in stakeholder interviews but not survey respondents. The stigma associated with a cervical cancer diagnosis led to some women becoming fearful about the possibility of negative societal reactions towards them due to perceived sexual promiscuity connotations.^37^ Indeed, as recounted by interview participants, some women withheld information about their diagnosis from supportive family and friends due to their concerns about becoming devalued as a direct result of a diagnosis. 221+ has used its position to raise awareness about the illness to good effect. Its continued advocacy and openness about the disease assist in representing women experiencing shame or in fear of becoming diminished in the eyes of others due to a cervical cancer diagnosis. Importantly, 221+ created a possibility for women to meet similarly situated others. This is an important support for women and its efficacy is endorsed by a growing body of research which highlights how those who have the benefit of in-group support experience reduced stress and enhanced well-being which collectively allows people to interpret significant life challenges as more manageable.^33^ In the context of these demonstrated benefits for women and their families, the continued presence and activity of 221+ in this area appears valuable.

### Implications for policy and practice

The project has several policy and practice implications which have immediate relevance in the Irish context but also, generate learnings for the international context where patient representation models in healthcare are being considered or are in operation.

First, 221+ offers support to women and families impacted by systems failures in CervicalCheck. The positive findings from interview and survey data suggest that the patient representative group should be more widely available to include *all* women diagnosed with cervical cancer, analogous to a service that up until recently existed in the United Kingdom known as Jo’s Trust (Jo’s Cervical Cancer Trust was an organisation in the United Kingdom (ceased trading on 23 May 2024) which raised awareness about cervical cancer and provided information and support to women and families impacted). 221+ provided a shared model of support for those with cervical cancer, and to date, the 221+ group is the only service in the Irish jurisdiction that provides a platform in which similarly situated others can meet and gain support.^32,34^ In addition, findings that many medical and health professionals were not familiar with 221+ points to a need for improvements in both outreach and awareness with both medical and health professionals and in public spheres, which has the potential to enhance the reach of 221+ as a platform of support for women impacted by cervical cancer.

Second, 221+ played a vital part in the support of patient representatives. Participants in this research recognised how patient representatives were an important presence in decision-making forums. However, difficulties in recruiting individuals willing to take such a responsibility were noted with barriers and costs to such work including, but not limited to, lack of clearly defined roles and responsibilities, financial implications and its time-consuming nature.^11,31^ 221+ however, can support patient representatives to ensure their contributions remain greater than a tokenistic symbol of PPI^31^ in healthcare forums. Additionally, the availability of a patient representative group has been noted as a crucial support scaffold for patient representatives to ensure the ‘patient voice’ is acknowledged in healthcare settings, that they are collaborators in decision-making forums and, importantly, they do not become overburdened with expectations or workloads.^10^

Third, the present research suggests that there is potential to include the ‘patient voice’ in educational courses with participants suggesting a role for 221+ patient representatives to target medical and health professional training programmes and schools to promote vaccination uptake. 221+ is well-placed to bring the ‘lived experience’ into educational settings to emphasise the importance of the patient voice in healthcare provision. In tandem, the group would start on a pathway towards a more collaborative partnership with medical and health professionals, which would also serve to both begin and enhance the restoration of trust processes.

Fourth, this research data emphasised the need for a restoration of trust between service users and healthcare services. 221+ is very well placed with the requisite skills and strengths, including strength in communicating and its ability to influence key stakeholders, to play an important role in these processes. The path towards building trust is a challenging one and highlights the need for the patient voice to be awarded an equal footing as a collaborator in decision-making forums.^11^

### Strengths, limitations and future directions

This article draws on evaluation gleaned from a small, purposeful sample of key stakeholders (interview respondents) and medical and health professionals (survey respondents). This two-phase qualitative design obtained a breadth and depth of data about the impact of the 221+ patient representative group across key stakeholder groups and those medical professionals women encounter on the ‘patient pathway’ for screening and/or treatment for cervical cancer. It was hoped that the qualitative data obtained from such varied sources might be beneficial in developing stronger communication bridges between medical and health professionals, patient representatives and service users. However, the findings may not be representative of views held by all medical and health professionals or stakeholders associated with the 221+ patient representative group. There is also a possibility of selection bias amongst survey respondents as those who participated may have been more motivated to engage with the research than others.

There is a future need for more large-scale research into both the experiences of medical and health professionals when harmful healthcare events occur to ascertain the support needed for those delivering patient services. Additionally, future research might consider the impact of the patient voice across cancer screening services in Ireland to determine the support needed by those patient supporters. In addition, future research should consider the longer-term impacts on patients and families who have experienced harm due to medical errors to establish their support needs over time. Finally, research might consider the stigma experiences of individuals living with cervical cancer and potential paths that might be taken to navigate through their stigma and the supports that have the potential to alleviate their shame of becoming diagnosed with cervical cancer.

## Conclusion

The research highlights the impact of patient representative group (221+) not only on supporting their members but also, more widely, on healthcare delivery in Ireland, particularly for women affected by cervical cancer. By evaluating the effectiveness of the 221+ group, the research provides valuable insights into how patient advocacy can be integrated into healthcare systems to ensure that patient voices are heard and acted upon. This is particularly relevant in the context of healthcare reforms worldwide, where there is a growing emphasis on transparency, accountability, and patient empowerment. The findings may inform the development of patient advocacy models globally, demonstrating how structured patient representation can influence healthcare policies and improve outcomes. The two-phase qualitative approach and reflexive thematic analysis used in the present research may serve as a methodological reference for other researchers and healthcare professionals interested in evaluating the impact of patient advocacy in other contexts. Importantly, this research contributes to the global discourse on women’s health, highlighting the importance of patient advocacy in addressing systemic issues and improving healthcare outcomes for women.

## Supplemental Material

sj-docx-1-whe-10.1177_17455057251351415 – Supplemental material for Evaluating the impact of a patient-representative model of support for women affected by cervical cancer Supplemental material, sj-docx-1-whe-10.1177_17455057251351415 for Evaluating the impact of a patient-representative model of support for women affected by cervical cancer by Elaine L. Kinsella and Elaine Kavanagh in Women’s Health
