# Supplementary material for: Evaluating the impact of a patient-representative model of support for women affected by cervical cancer
Source: Womens Health (Lond). 2025 Jul 18;21:17455057251351415. doi: 10.1177/17455057251351415 (PMC12276518; doi:10.1177/17455057251351415)
Supplement: sj-docx-1-whe-10.1177_17455057251351415 – Supplemental material for Evaluating the impact of a patient-representative model of support for women affected by cervical cancer [file sj-docx-1-whe-10.1177_17455057251351415.docx]

**221+ Interview schedule**

The purpose of the interview is to establish the key activities and associated impact of the organisation of 221+ from the perspectives of different stakeholders.

1. What is your own connection to the 221+ Patient Support Group?
2. How familiar are you with the work of 221+ Patient Support Group as an organisation?
3. What activities have 221+ facilitated or contributed to, in your view, that have been important?
4. How has 221+, as an organisation, impacted i) those with cervical cancer that was not identified through screening and their families, ii) the healthcare system in Ireland iii) Irish or international politics, and iii) and Irish society and culture [including financial/economic, institutional]? *Follow up:* What, in your view, have been the most significant contributions of 221+ as an organisation? Are there any other impacts that you haven’t covered yet?
5. How can 221+ build on previous work to improve women’s healthcare in Ireland?
6. What, in your view, have been the missed opportunities (if any) for 221+ as an organisation?
7. In what other ways can 221+ contribute to Irish society in the future, particularly in relation to women’s healthcare and patient voice?
8. We are coming close to the end of the interview now. Is there anything else that you would like to add?

**Survey schedule**

**The purpose of this short survey is to establish a range of views of the organisation of 221+ and patient representatives from the perspectives of medical and health professionals in Ireland.**

*The 221+ Patient Support Group was established in July 2018 to provide information, advice, and support to the women and families directly affected by failures in the CervicalCheck Screening Programme that came to light following Vicky Phelan’s court case in April 2018. It is a completely independent organisation and is not operated by the HSE or Department of Health.*

1. What do you know about the work of 221+ Patient Support Group in Ireland?

2. In your view, what have been the key activities and/or contributions made by 221+ Patient Support Group?

3. What, in your view, have been the missed opportunities of 221+ Patient Support Group as an organisation?

4. How has the work of 221+ Patient Support Group impacted your work life and/or personal life?

5. In what ways do you think 221+ Patient Support Group could contribute to Irish society in the future?

**Before completing the survey, please answer the following demographic questions:**

**6. Please tick the box that best represents your age:**

  ☐ 18 – 30 years old

  ☐  30 – 45 years old

  ☐  45+

  ☐  Prefer not to answer

**7. Please tick the box that best represents your gender identity:**

  ☐ Woman

  ☐ Man

  ☐ Transgender

  ☐ Non-binary/non-conforming

  ☐ Prefer not to respond.

**8. Please tick the box that best represents your occupation:**

  ☐ Health professional

  ☐ Health associate professionals

  ☐ Personal care workers in health services

  ☐ Health management and support personnel

  ☐ Other health service providers

**9. State your occupation here (optional):**
